# Should Examining Boards Have Power to Grant Certificates of Qualification to Undergraduates?

**Published:** 1893-01

**Authors:** Lewis Jack


### SHOULD EXAMINING BOARDS HAVE POWER TO GRANT CERTIFICATES OF QUALIFICATION TO UNDERGRADUATES?[1]

BY LEWIS JACK, D.D.S.

The question is one of extreme consequence to the cause of dental education, and demands from us and from any body of dentists essaying to discuss it the most careful, exhaustive, and judicious consideration. The wording of this question probably does not go sufficiently far. It should have included in its

---

[1] Read before the Odontological Society of Pennsylvania, November 18, 1892.

verbiage the further inquiry whether the protective influence of law should cover any form of qualification for practice except that which is given by compliance with the fullest curriculum of the dental schools. In other words, the term undergraduate should include every one who is not a graduate of a dental school. Such was the intention in the mind of the proposer of the question.

Therefore my argument before you in opening this discussion will include both classes of those who may apply to the Examining Boards, and those who have been self-taught, and those who have attended one or two courses of instruction.

I have examined the laws of the various States, principally as they are reported in Rehfuss's "Dental Jurisprudence," and find the following States give any person the right without qualification to demand an examination as to their attainments,—viz., Alabama, California, Colorado, Delaware, Georgia, Illinois, Indiana, Iowa, Louisiana, Maine, Massachusetts, Michigan, Mississippi, North Carolina, Oklahoma, Pennsylvania, Rhode Island, South Carolina, Tennessee, Texas, Vermont, Virginia, Washington, West Virginia, Wisconsin.

The following States qualify this privilege by requiring evidence of previous study: New Jersey makes five years' preparation a necessity. New York if a "period of four years of regular study has been pursued." North and South Dakota each require "three years' previous study under the supervision of some regularly practising dentist."

The States which do not examine such persons are Arkansas, Kansas, Kentucky, Maryland, Minnesota, Missouri, Nebraska, and Ohio.

It is thus to be observed that the laws of twenty-eight States permit the examination of non-graduates, and that those of eight States do not authorize the examination of persons unqualified by the various schools.

The fundamental principle concerned in the requirement that the Examining Boards may give certificates to practise to those who are not regularly qualified, is based on the probability that there may be some who have made the necessary preparation to render intelligent service, and who may not have enjoyed the advantages of school training, and that, therefore, these should not be excluded from the enjoyment of the inherent right of man to freedom in the pursuit of business. The probability of the existence of such a class of persons, even in the most intelligent communities, is so remote that the injury caused by the relinquishment by the public of the right of any person to an examination is slight in comparison with the damage which may be inflicted by its retention. This being so, there can be no great importance to be attached to the existence of this privilege.

From the experience of the past, the liability to leniency of the Examining Boards granting certificates of qualification to indifferently prepared persons is a constant menace. It is a source of contention by the teaching Faculties and a ground of contempt for the boards. The fault is in the law, of which the boards are the executors; but the fact remains that Examining Boards invest men with the rights which the Faculties consider three years of exhaustive study as no more than sufficient to prepare them to enjoy.

The general purpose of the dental laws, which have become of nearly universal enactment, have for their first effect the protection of the public from the injuries which may be inflicted upon the members of it by incompetent persons. The immediate secondary result is to induce those contemplating the pursuit of dentistry to make the necessary effort and personal sacrifice of time and money to pass through the required steps to secure thorough training. The result of the latter force is what has been in the minds of those who have proposed and brought about the approval of the dental laws. For this reason these laws have been a benefit to the public, an unmeasurable advantage to the schools, and have proved immensely beneficial to the character of dentistry as a liberal pursuit.

The presence of the principle, however, that any person may come forward and submit to an examination has had a demoralizing effect upon many who, without this opportunity, would have had no other course open than either to undergo a college preparation or abandon the attempt.

No one can indicate how many have in the various States carried on their attempts to practise with the vain hope that they could prepare themselves for examination, and have in the mean time gone on surreptitiously practising when there appeared little danger of being disturbed by their neighbors.

My views upon the next to impossibility of any person of the class indicated making a creditable examination have been long entertained, and have become strengthened by my experience on the Examining Board of this State. In the two terms I acted not a candidate could, on my subjects, answer correctly the most elementary questions. For instance, on the subject of dental histology, I have yet to get an approximately correct reply to the question, "What is the character of the structure of the mucous membrane?" Of course the genesis of tooth-tissue can have no place in the mind of a student who cannot answer this query. In dental anatomy I have yet to receive a satisfactory definition of the three hard structures which enter into the teeth. From my examination of such candidates I have been forced to the conclusion that the examination is simply useless, and that the requirement that boards shall entertain their demands is a waste of time. It is objectionable because it is unreasonable in the face of the facts; it borders on the absurd because it is fruitless.

In too many instances, when persons of this class have been given the certificate to commence or to continue practice, they have been an injury to the neighboring practitioners in ways scarcely necessary to recount here.

There remains the further question of the injustice of such persons being admitted to practice. The college student who has spent his two or more years at a large expense in a distant city for qualification finds himself confronted by another who has the same rights under the law, who at a few dollars' outlay has procured this privilege and protection, and who, not having the air of professional honor about him, has poisoned the atmosphere in various ways by degrading methods of conducting practice, accompanied in some instances with the pretension that the certificate gave greater claims because it was the result of an examination by a board of men under the authority of the State and the auspices of the State Dental Society.

This much being stated should be sufficient to conclude the question of the impropriety of this qualifying process.

While it may have had some justification in the early legal regulation of dentistry, since at that time it would have been difficult to procure legislation without granting this right, it certainly is not in consistency with the views of leading men. The new laws as they are amended should withdraw this privilege. That it may be done is conclusively proven by the fact that, as alluded to above, eight States require graduation as a qualification to enter practice; and as no legal question upon this point has arisen, so far as we are aware, it may be taken for granted that no impediment has been encountered.

When we come to another class of persons, those who have attended two years of school training, who may be offering themselves ere long in the States which are required to examine any who may present for certificates, the discussion assumes a still more serious aspect.

The expression of opinion by leading dentists that the curriculum of the dental schools required enlargement and the period of study extension, with the concurrent action of the schools and the Association of Dental Faculties to the same effect, resulted in improved courses of study in some of the schools, and the enforcement by the Association of Faculties upon their associates of the three-years' term of instruction. This enlargement of the course of study had the support and encouragement of the National Board of Examiners, and marks an important advanced step in dental education. The danger of this action being weakened by the obligation of the Examining Boards in so many States to make examination of any undergraduate who may present for this test, makes the question now before us an extremely important one. It is readily perceived that it will tend to weaken the cause of dental education, and is a menace to the three years' course of instruction. How far students who have completed the second year may attempt to shorten their period of instruction by resorting to an attempt to procure certificates of qualification by applying to the boards for an examination is an uncertain factor, but the probability of such a course, even the possibility of it, should occasion alarm to the friends of extended dental education. The opportunity for the accomplishment of such a result is a menace, and needs the correcting influence of public opinion to remove the chances.

The general tendency and the undercurrent force of the dental enactments regulating the practice of dentistry is to bring about improvements in the dental curriculum, to increase the importance of dental services and enlarge the self-respect of those engaged in practice. In view of these important ends which are involved, it is extremely illogical to entertain the propriety of examining undergraduates in the manner indicated. Such a course would be begging the whole question of dental education, and implicates every one who is concerned as a participant in curtailing the benefits to which every honorable man, who regards the good of his profession, is committed.

The injurious effects upon the morale of the dental profession would appear a greater evil than the examination of mere novices.

It would produce distrust of the Examining Boards upon the part of the Faculties, would weaken the integrity of the individual offering himself for examination, and being so inconsistent with the tendency of the times which demands more exacting preparation, that mental confusion and moral distress must be the inevitable result.

Examining Boards may have an understood policy which may render it difficult for any one to pass their ordeal, but it appears there can be no safety for the schools of the dental profession until the laws are modified to make it impossible to get a certificate of qualification from a Board of Examiners alone.

As the schools are now entering upon the regular three years' course of study, there is abundance of time for the various State societies to make application for such amendments of the dental laws as will repeal the privilege of examining non-graduates.

Certainly it cannot be too much for this Society to express its sentiments to the Pennsylvania State Dental Society, through its committee on dental laws, that the new or amended law about being prepared for approval in Pennsylvania should include such provision as will remove the privilege to grant certificates of qualification to any person who is not a graduate.

A similar request sent to the officers of the various State Dental Societies would also be of service to the same end.

This subject will be presented to the many local societies for discussion in the same form it has been given to this Society, and while it is a fresh topic is the proper time to give it prominence by an endeavor to produce concurrent action in the States which will benefit by the proposed amendment.

